# Identification of a novel 2-oxindole fluorinated derivative as in vivo antitumor agent for prostate cancer acting via AMPK activation

**DOI:** 10.1038/s41598-018-22690-2

**Published:** 2018-03-12

**Authors:** Alicia Bort, Sergio Quesada, Ágata Ramos-Torres, Marta Gargantilla, Eva María Priego, Sophie Raynal, Franck Lepifre, Jose M. Gasalla, Nieves Rodriguez-Henche, Ana Castro, Inés Díaz-Laviada

**Affiliations:** 1 Department of Systems Biology, School of Medicine, University of Alcalá, Alcalá de Henares, E-28871 Madrid, Spain; 2 Instituto de Química Médica (IQM-CSIC), Juan de la Cierva 3, E-28006 Madrid, Spain; 3 Metabrain Research, 4 Ave. du Pdt. François Mitterrand, 91380 Chilly Mazarin, France; 4 Clinical Biochemistry Service, Principe de Asturias Hospital, Alcalá de Henares, E-28871 Madrid, Spain; 5 Chemical Research Institute “Andrés M. del Río” (IQAR), University of Alcalá, Alcalá de Henares, 28871 Madrid, Spain

## Abstract

The key metabolic sensor adenosine monophosphate-dependent kinase (AMPK) has emerged as a promising therapeutic target for cancer prevention and treatment. Besides its role in energy homeostasis, AMPK blocks cell cycle, regulates autophagy and suppresses the anabolic processes required for rapid cell growth. AMPK is especially relevant in prostate cancer in which activation of lipogenic pathways correlate with tumor progression and aggressiveness. This study reports the discovery of a new series of 2-oxindole derivatives whose AMPK modulatory ability, as well as the antitumoral profile in prostate cancer cells, was evaluated. One of the assayed compounds, compound **8c**, notably activated AMPK in cultured PC-3, DU145 and LNCaP prostate cancer cells. Likewise, compound **8c** caused PC-3, DU145 and LNCaP cells viability inhibition. Selective knocking down of α1 or α2 isoforms as well as *in vitro* assays using human recombinant α1β1γ1 or α2β1γ1 AMPK isoforms revealed that compound **8c** exhibit preference for AMPKα1. Consistent with efficacy at the cellular level, compound **8c** was potent in suppressing the growth of PC-3 xenograft tumors. In conclusion, our results show that a new 2-oxindole fluorinated derivative exerts potent *in vivo* antitumor actions against prostate cancer cells, indicating a promising clinical therapeutic strategy for the treatment of androgen-independent prostate cancer.

## Introduction

In the last decade, growing evidence about tumors origin and progression has allowed to suggest new additional hallmarks of cancer, which may be involved in the pathogenesis of some and perhaps all types of this disease. One of them involves the capability to modify, or reprogram cellular metabolism, in order to support high growth rate and energy requirements that are essential for the sustained tumor growth and progression^1–3^. In particular, a hallmark of prostate cancer is the increased de novo fatty acid and cholesterol synthesis which correlates with tumor progression and poorer prognosis^4–6^. Thus, targeting these upregulated pathways with inhibitors of key enzymes and associated molecular regulators or by downregulation of lipogenic genes may be a favorable strategy whereby the properties of prostate cancer cells can be exploited for therapeutic gain. In this context and considering the role of the AMP-activated protein (AMPK) as a sensor of cellular enery status, the AMPK modulation appears as a promising approach to develop new therapeutic strategies for cancer prevention and treatment^7–9^. Nevertheless, the role of AMPK in prostate cancer still remains controversial as recent research suggests that AMPK can exert pro- or anti-tumorigenic roles in cancer depending on context^10^. Several studies have shown that AMPK activation by metformin, MT63–78 or LKB1 overexpression suppresses prostate cancer cells viability and reduces their metastatic properties^11–13^. Authors demonstrate that the suppression of de novo lipogenesis is the underpinning mechanism responsible for AMPK-mediated prostate cancer growth inhibition^12^. In addition, blockage of fatty acid synthesis by inhibition of stearoyl-CoA desaturase (SCD)^14^ or fatty acid synthase^15^ suppresses prostate cell survival. Likewise, expression and activity of LKB1 has also been inversely related with proliferation and survival of prostate cancer and prostate cancer cells^16,17^.

The potential of targeting AMPK activation in cancer prevention has been further supported by epidemiological studies in type 2 diabetes patients revealing the ability of metformin to reduce cancer risk and recurrence^18–20^. Moreover, *in vitro* and *in vivo* studies have demonstrated that metformin potentiates the effect of chemotherapeutic drugs and produce synergistic effects on efficacy against prostate cancer^21–23^. In contrast, recent analyses in diabetic patients did not show relationship between metformin and prostate cancer protection^24^. Hence, the functional role of AMPK in prostate cancer is much more complex than expected as a role for AMPK as a survival pathway for cancer cells has also been demonstrated^10^.

Until now, many efforts from industrial and academia have been focused on searching novel agents that modulate AMPK directly or indirectly. From a drug discovery program perspective, crystallographic studies of full-length and truncated AMPK heterotrimeric complexes have provided important insights related to regulatory mechanisms and also about the potential binding mode of AMPK modulators^25,26^. In this context, A-769662, one of the better-characterized AMPK activator, or the indole, PF-06409577 (**1**), recently reported by Pfizer for the treatment of diabetic nephropathy, appear as some representative examples^27^.

**Figure 1:**
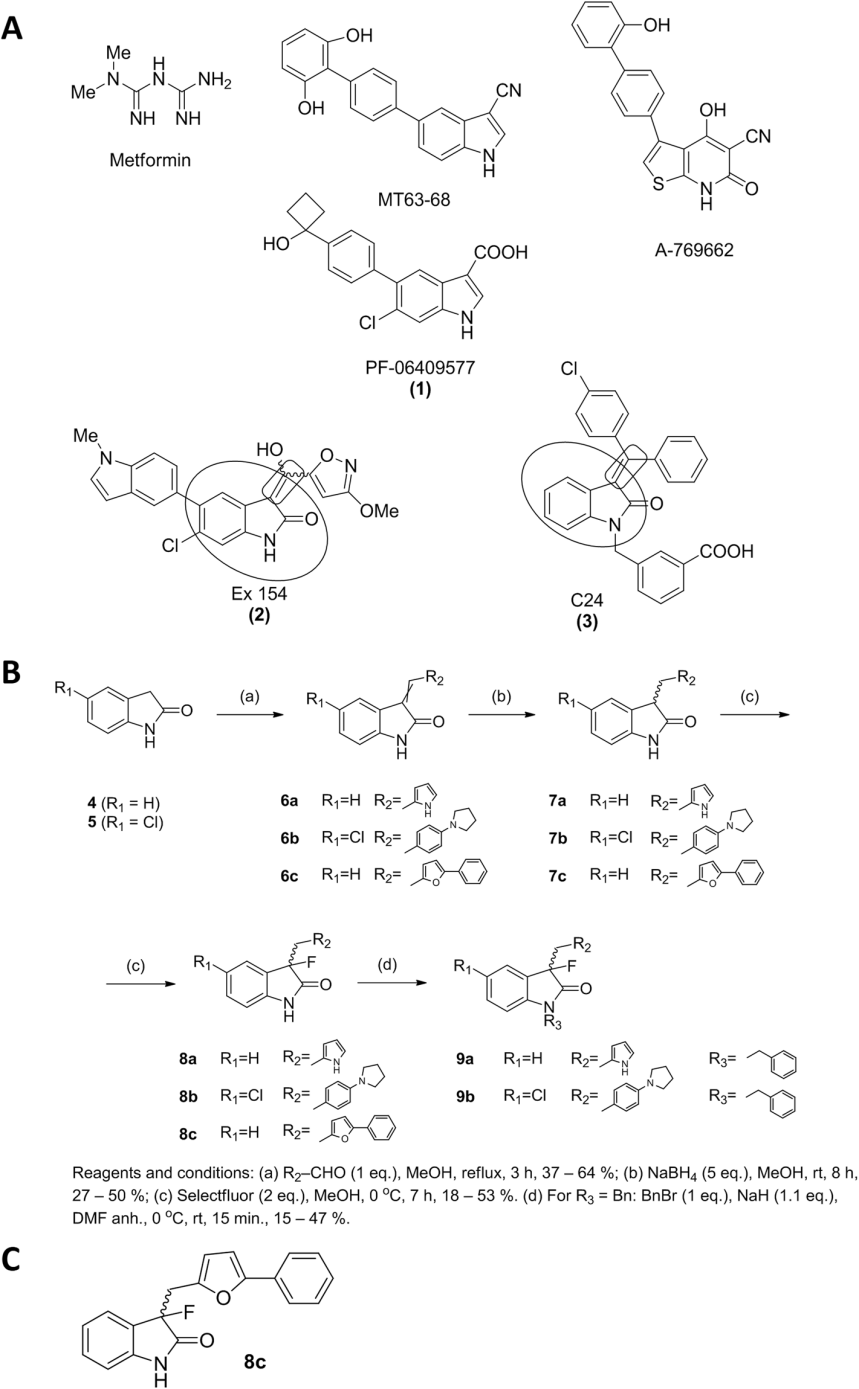
(**A**) Structures of selected AMPK modulators. (**B**) Synthetic route followed for the synthesis of final compounds. (**C**) Structure of the compound **8c**.

In this regard, oxindole derivatives have emerged as privileged scaffold in the AMPK modulation. As examples, EX154 (**2**) or C24 (**3**) are established as potent AMPK activators for the treatment of several diseases^28,29^ (Fig. 1A). These families share some structural features, which may be important for AMPK activation. Both representative examples show an 2-oxindole moiety with a alkene substitution at C-3 position respect to indole system. On the basis of these findings, we considered 2-oxindole moiety as an interesting chemical scaffold to explore the way of modulating AMPK and its role in prostate cancer.

Based on the general overview of the 2-oxindole structures previously described (Fig. 1A), we propose that C-3 substituents may presumably play a determining role for the biological action. In particular, we paid attention to C24 (**3**) compound in order to propose novel prototypes. Thus, we proposed the replacement of the double bond at C-3 of 2-oxindole scaffold by a methylene linker, as main structural novelty. Therefore, we will allow us to determine the impact of this flexible modification on AMPK modulation.

## Results

### Chemistry

The 2-oxindole derivatives were prepared following the synthetic route depicted in Fig. 1B. Compounds **6a-6c** were synthesized by condensation of commercially available 2-oxindole derivatives (**4** and **5**) with different aromatic aldehydes^30^. Reduction of **6a-6c** was achieved with sodium borohydride in methanol to result **7a-7c** in 37–64% yield. The subsequent fluorination reaction placed a fluorine substituent at C-3 position on the 2-oxindole skeleton, which is easily performed using compounds **7 a-c** with Selectfluor, as electrophilic fluorine source, to yield the novel compounds **8 a-c**^31,32^. Finally, N-alkylation using commercially available alkylation agents was achieved in anhydrous DMF using NaH as a base to led the final derivatives **9a-9b** with moderated yields^33^.

### Evaluation on AMPK activity in prostate cancer cells

**Figure 2:**
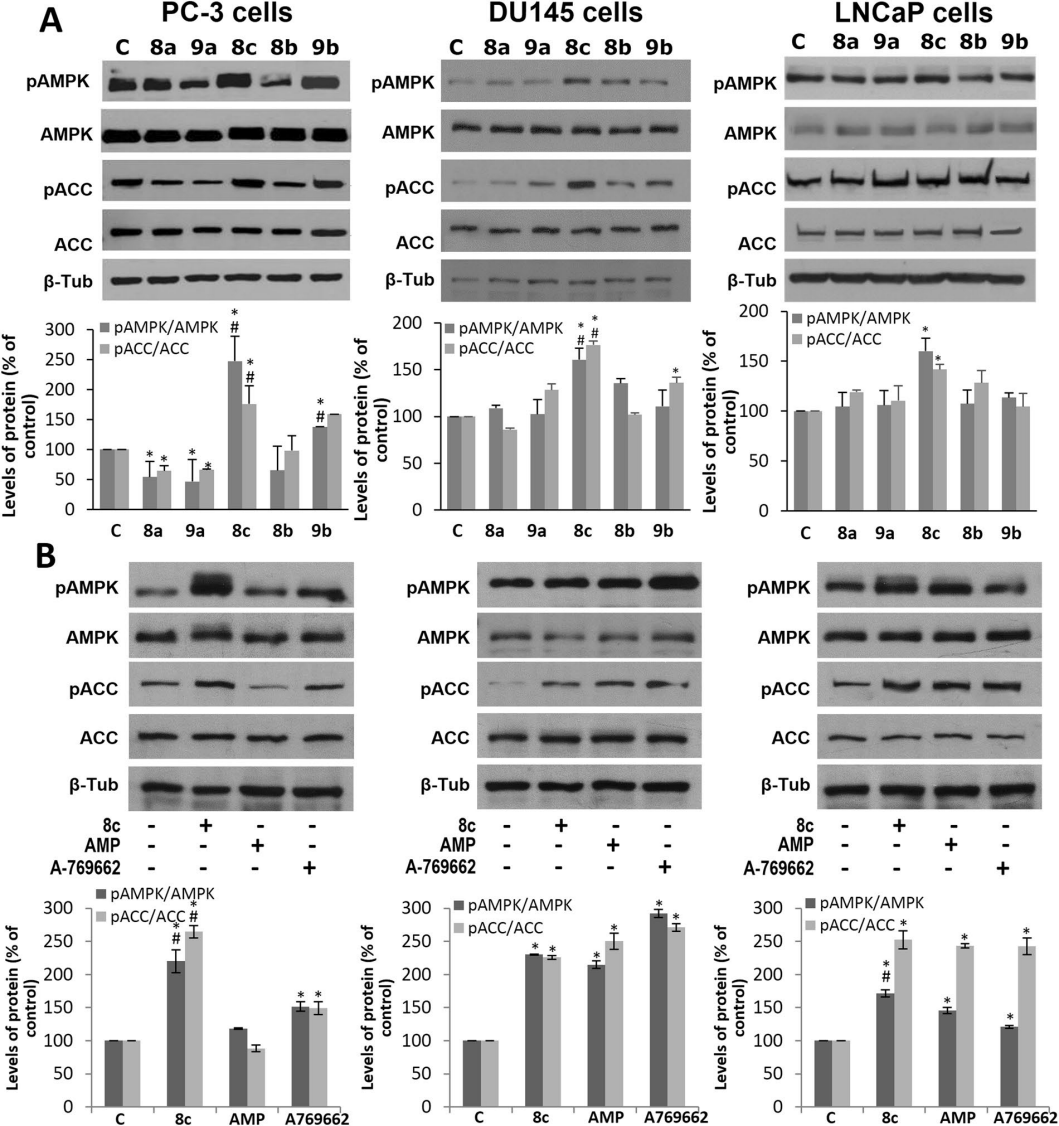
Effect of 2-oxindole fluorinated derivatives on AMPK activity in prostate cancer cells. (**A**) PC-3, DU145 and LNCaP cells were treated with 25 µM of the selected 2-oxindole fluorinated derivatives for 1 hour. Levels of the phosphorylated AMPK (pAMPK), phosphorylated ACC (pACC) and their total forms were determined by Western blot. Upper panel, a representative Western blot of three different experiments. β-tubulin (β-Tub) serves as a loading control. (**B**) PC-3, DU145 and LNCaP cells were treated with 25 µM of compound **8c**, 25 µM of AMP or 25 µM of A-769662 for 1 hour and processed as above. Lower panels, densitometric analyses of bands represented as the mean of the ratio pAMPK/AMPK or pACC/ACC ± SD of three different experiments. *p < 0.05 compared with the controls by one-way ANOVA followed by Fisher’s LSD test; ^#^p < 0.05 compared with 2-oxindole fluorinated derivates or with the AMPK activators AMP or A769662 by one-way ANOVA followed by Tukey test.

To investigate the effect of the compounds on AMPK activity at cellular level, compounds **8a-c** and **9a-b** were selected to perform AMPK phosphorylation assays in human prostate cancer cell lines. We use the PC-3 cell line as it is androgen-resistant and represents the advanced and aggressive stage of prostate cancer. We found that compounds **8a**, **9a** and **8b** slightly, although significantly, inhibited AMPK phosphorylation compared to controls and compound **9b** exerted a weak AMPK activation. In contrast, compound **8c** caused a significant increase in Thr172 phosphorylation of AMPK compared to control cells (Fig. 2A). AMPK inhibits lipogenic pathway through phosphorylation of its well-known substrate acetyl-CoA carboxylase (ACC). To confirm the modulation of AMPK activity in prostate cells by the compounds, we determined levels of phosphorylated ACC in 2-oxindole fluorinated derivatives-treated cells. As shown in Fig. 2, levels of pACC correlated to the levels of pAMPK. Whereas compounds **8a**, **9a** and **8b** inhibited ACC phosphorylation, compounds **8c** and **9b**, according to their increased AMPK activation, augmented pACC. It is worthy to note that the highest ACC phosphorylation was obtained with compound **8c** denoting a powerful activity of this compound in PC-3 cells.

To further confirm the effect of the compounds in prostate cells, we used the androgen-insensitive cell line DU145 in which LKB is inactive and the androgen-sensitive prostate cancer LNCaP cell line. Similar results were obtained although AMPK stimulation by compound **8c** was not so strong (Fig. 2A). The fact that compound **8c** stimulate AMPK in DU145 cells suggest that LKB1 is not necessary for **8c** effect and points to a direct effect of **8c** on AMPK.

To corroborate the activation of AMPK by compound **8c**, it was compared with the two well-recognized AMPK activators AMP and A-769662 which bind to the regulatory subunits and allosterically activate the kinase activity of the AMPKα^34^. As shown in Fig. 2B, compound 8c induced AMPK and ACC phosphorylation comparable to AMP or A-769662 (Fig. 2B).

### Effect of 2-oxindole fluorinated derivatives on prostate cancer cells proliferation

**Figure 3:**
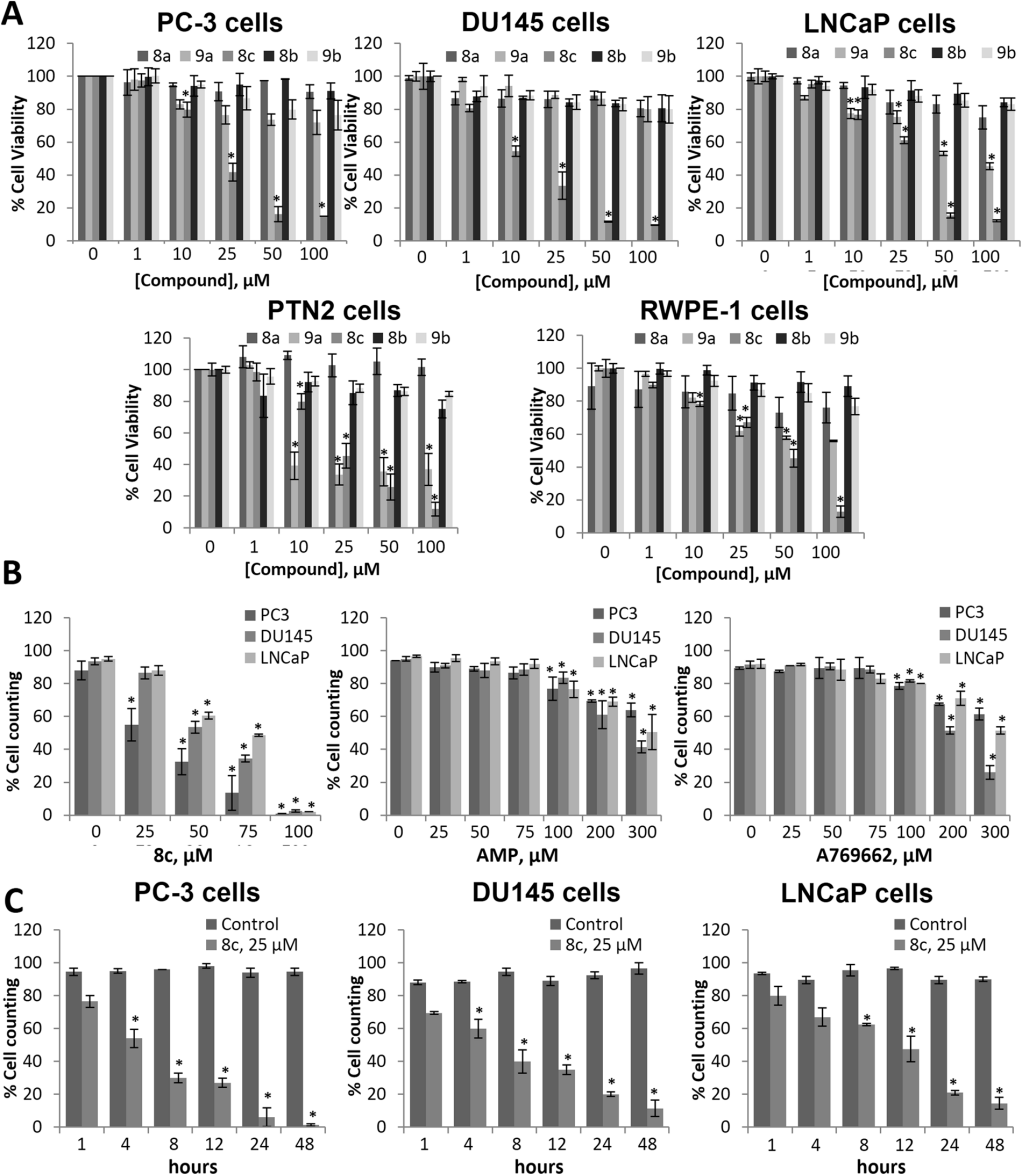
Comparison of the effect of 2-oxindole fluorinated derivatives on the proliferation of human prostate cancer cells. (**A**) Normal (PTN2 and RWPE-1) and cancer (PC3,DU145 and LNCaP) prostate cells were treated with different doses of the compounds for 24 hours and cell viability was monitored by MTT assay. (**B**) PC-3, DU145 and LNCaP cells were treated with different concentrations compound **8c**, AMP or A-769662 for 24 hours, and then viable and total cells were counted. (**C**) PC-3, DU145 and LNCaP cells were treated with 25 µM of compound **8c** for different times. Values were normalized with the control and represent the mean ± SD of at least three independent experiments. *p < 0.05 and **p < 0.01 compared to control by the Student’s - test.

We then study the effect of the compounds on the proliferative capacity of PC-3, DU145 and LNCaP prostate cells. We also used the prostate cell lines PTN2 and RWPE-1 which are derived from a histologically normal adult and therefore are considered as normal prostate cells, for comparison. All the compounds were tested at concentrations ranging from 1 to 100 μM. We found that compounds **9a** and **8c** effectively inhibited cell proliferation in the range of 1–100 µM in a dose-dependent manner in the five cell lines (Fig. 3A), although compound **8c** exhibited a clear antiproliferative effect. Notably, compound **8c** exhibited a greater efficacy and potency against the cancer cell lines LNCaP, DU145 and PC-3 compared to normal cells (Fig. 3A).

In fact, the highest potency of this compound is observed in the androgen-resistant PC-3 cells, which represent the most aggressive form of prostate cancer. IC50 values for compound **8c** were 18 μM, 19 μM, 30 μM, 65 μM, and >1000 μM in PC-3, DU145, LNCaP, PNT2 andRWPE-1, cells, respectively. As shown in Table 1, at 50 μM compound **8c** was the most efficacious in all cell lines displaying a higher antiproliferative effect against the cancer cell lines PC-3, DU145 and LNCaP. Nevertheless, at 100 µM, compound **8c** almost totally inhibited cell viability in the five cell lines probably due to a high cytotoxic effect (Fig. 3A).

**Table 1:** Comparison of inhibitory efficacy of 2-oxindole fluorinated derivatives on human prostate cancer cells viability at 50 μM.

	PTN2	RWPE-1	LNCaP	PC-3	DU145
**8b**	0	17%	11%	3%	14%
**9b**	52%	42%	47%	27%	14%
**8c**	56%	50%	85%	84%	88%
**8d**	14%	8%	11%	2%	16 €
**9b**	8%	8%	10%	23%	17%

These data indicate that the highest inhibitory effect correlates with the greater activation of AMPK in prostate cancer cells and prompted us to continue the study with the compound **8c**, (structure in Fig. 1C) being selected for further studies.

To confirm the antiproliferative effect of compound **8c**, cell viability was evaluated by cell counting at different times in PC-3, DU145 and LNCaP cells. As observed in Fig. 3B, **8c** induced a time-dependent strong cytotoxic effect in the three cell lines although it was more pronounced in PC-3 cells.

The inhibitory efficacy of **8c** in prostate cells was contrasted with that of AMP and A-769662 by cell counting and results indicate that compound **8c** is very potent and effective especially in the PC-3 cell line (Fig. 3B).

**Figure 4:**
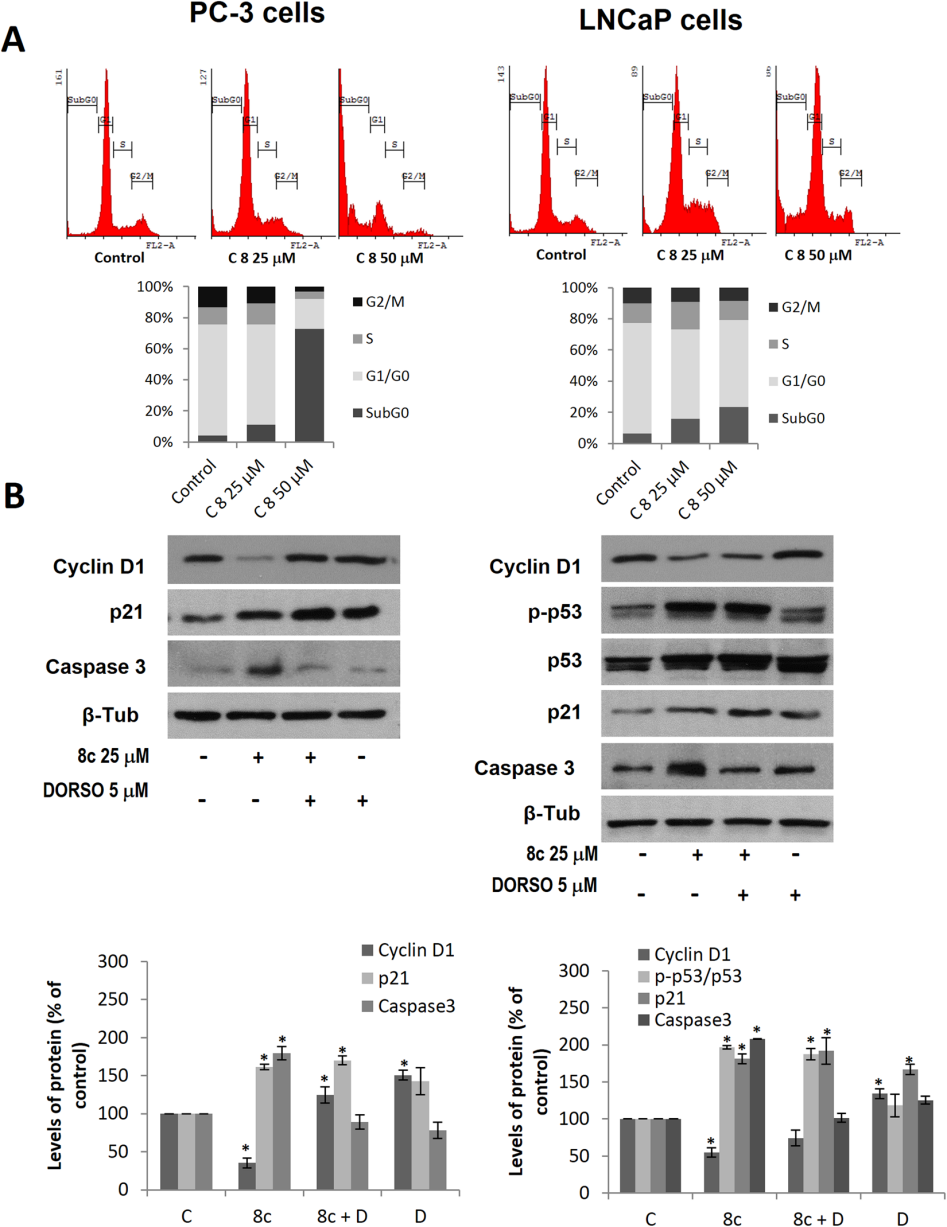
Cell Cycle analysis in 8c-treated prostate cancer cells. (**A**) PC-3 and LNCaP cells were treated with compound **8c** for 24 hours and cell cycle analysis was analyzed by flow cytometry using Propidium Iodide (PI). Percentage of cells in subG0, G1, S, and G2/M phases is indicated in the histogram. (**B**) Levels of Cyclin D1, p-p53, p53, p21 and cleaved Caspase 3 (17 kDa fragment) were determined by Western blot. Upper panel, representative Western blot of two different experiments. β-tubulin (β-Tub) serves as a loading control. Lower panel, the densitometric analyses of bands represented as the mean ± SD of the two different experiments. *p < 0.05 and **p < 0.01 compared with the control group by the Student’s t-test.

Upon demonstration that compound **8c** strongly inhibited prostate cancer cells growth, we investigated its effect on cell cycle. For that, we treated PC-3 and LNCaP cells with 25 μM and 50 μM of compound **8c** for 24 h and performed cell cycle analysis by flow cytometry using Propidium Iodide (PI). As shown in Fig. 4 an increase in subG0 cells was detected in cells treated with the compound **8c**, fact that indicates apoptosis. It is worthy to note that when PC-3 cells were treated with compound **8c**, 80% of cells were in subG0 phase (Fig. 4A).

There are many intracellular mechanisms whereby AMPK activation inhibits cell growth. Recent findings reveal that one possible mechanism is the downregulation of Cyclin D1 and cell cycle arrest^35–38^. Cyclin D1 senses and integrates extracellular signals and intracellular changes of cells in G1 phase and therefore its modulation reflects intracellular status. To further investigate the mechanism underpinning the cell viability inhibitory effect of compound **8c**, levels of Cyclin D1 were determined by Western blot in PC-3 and LNCaP cells. As shown in Fig. 4B, a marked decrease in Cyclin D1 level was observed in **8c**-treated cells. The decrease was prevented by incubation with the AMPK inhibitor Dorsomorphin (also known as Compound C), which has been shown to inhibit AMPK on prostate cells^39^, pointing to an AMPK-dependent effect (Fig. 4B).

Cyclin D1 activity is modulated by several inhibitors such as p21 which negatively regulate cell cycle progression by interacting with cyclin dependent kinases (cdks). P21 protein plays a crucial role in the signaling pathway of the tumor suppressor gene p53, which induces growth arrest and apoptosis when DNA is damaged. To further study the role of compound **8c** in cell cycle, we determined the expression of p21, p53 and the levels of cleaved caspase 3 in the prostate cells. P53 was only determined in LNCaP cells since PC-3 cells are p53-negative^40^. When cells were treated with **8c**, a remarkably increase in p21 protein was observed both in PC-3 and in LNCaP cells (Fig. 4B). In LNCaP cells, and increase in the total and phosphorylated forms of p53 was also observed. These changes correlated with an increase in cleaved caspase 3 (17 kDa fragment) indicating a cell cycle arrest and apoptosis induction by compound **8c** in prostate cells (Fig. 4B).

### Characterization of AMPK activation by compound 8c

**Figure 5:**
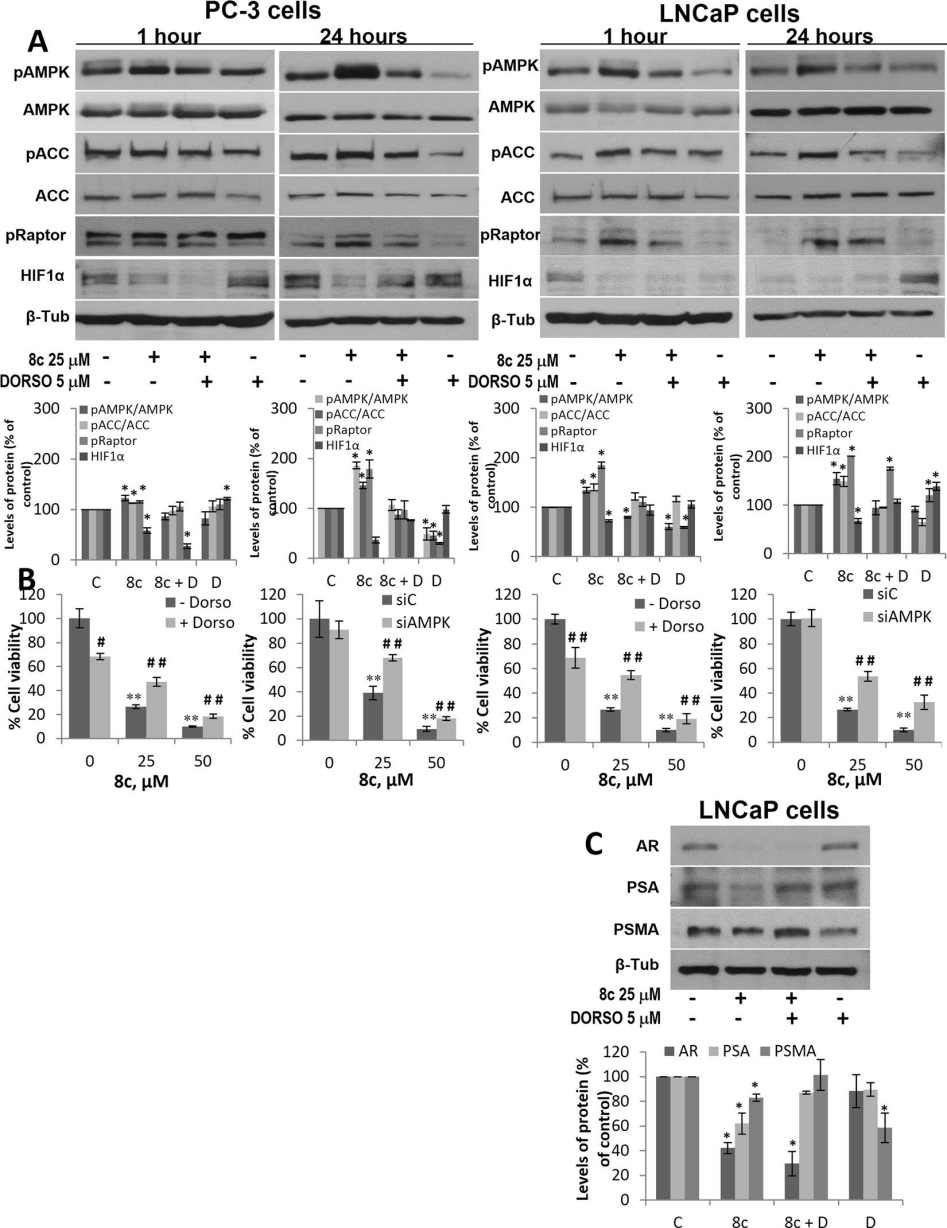
AMPK activation by compound 8c in prostate cancer cells. (**A**) PC-3 and LNCaP cells were treated with vehicle, 25 µM compound **8c**, 25 µM compound **8c** + 5 μM Dorsomorphin (8c + D) or 5 μM Dorsomorphin (D), for 1 hour or 24 hours and levels of the phosphorylated AMPK (pAMPK), phosphorylated ACC (pACC), phosphorylated Raptor (pRaptor) forms and HIF1α were determined by Western blot. Upper panel, representative Western blot of three different experiments. β-tubulin (β-Tub) serves as a loading control. Lower panel, densitometric analyses of bands represented as the mean of the ratio pAMPK/AMPK or pACC/ACC. (**B**) Cell viability monitored by MTT assay in PC-3 and LNCaP cells treated with vehicle, 25 µM and 50 μM compound **8c** alone or in the presence of 5 μM Dorsomorphin or transfected with control siRNA (siC) or α1AMPK-selective siRNA (siAMPK). (**C**) LNCaP cells were treated with vehicle, 25 µM compound **8c**, 25 µM compound **8c** + 5 μM Dorsomorphin (8c + D) or 5 μM Dorsomorphin (D), for 24 hours and levels of Androgen Receptor (AR), Prostate specific antigen (PSA) and Prostate specific membrane antigen (PSMA) were determined by Western blot.Values were normalized with the control and represent the mean ± SD of at least three independent experiments. *p < 0.05 and **p < 0.01 compared to control and ^#^p < 0.05 and ^##^p < 0.01 compared with **8c**-treated group by the Student’s t-test.

To further confirm the compound **8c**-induced activation of AMPK, PC-3 and LNCaP cells were incubated with the compound for 1 h and 24 hours in the presence of the AMPK inhibitor Dorsomorphin. As shown in Fig. 5A, activation of AMPK by compound **8c** was also seen at 24 hours, being even more robust that at 1 hour of treatment. Dorsomorphin blocked AMPK activation by compound **8c** both at 1 h and 24 h. Accordingly, ACC phosphorylation was increased at 1 h and 24 h and prevented by Dorsomorphin in both cell lines. AMPK inhibits mTORC1 through phosphorylation of Raptor on two well-conserved serine residues (ser722 and ser792) inducing cell-cycle arrest in response to an energy stress. To further support the **8c**-induced AMPK activation, we determined Raptor phosphorylation on Ser792 in prostate cells. An increase in p-Raptor was observed in PC-3 and LNCaP cells treated with **8c** which was partially prevented by Dorsomorphin (Fig. 5A). It has been demonstrated that AMPK activation suppresses the hypoxia-inducible factor 1 A (HIF-1A) accumulation induced by hypoxic stress, counteracting the inflammatory response and the HIF-1A-promoted tumorigenesis. As shown in Fig. 5A, the expression of HIF-1A was decreased in cells treated with compound **8c**. These results reinforce the notion of the **8c** antitumoral role through AMPK activation in prostate cells.

To analyze whether AMPK was involved in the antiproliferative effect of compound **8c**, AMPK pharmacological inhibition as well as AMPK knocking down were carried out and cell viability was assayed. Figure 5B shows that the decrease in cell viability induced by compound **8c** in PC-3 and LNCaP cells was reduced in cells treated with the AMPK inhibitor dorsomorphin. Likewise, AMPK silencing by specific siRNA, prevented **8c**-induced cell death, further supporting the involvement of AMPK in the antiproliferative effect of compound **8c**.

The androgen receptor (AR) plays a key role in prostate cancer progression and, as a result, targeting of AR signaling is a major therapeutic strategy. Recent findings reveal that AR is a potential downstream mediator of AMPK in LNCaP cells and that AMPK activation inhibited AR activity and translocation to the nucleus^41^ Therefore we analyzed the expression of the androgen receptor as well as of its two well-recognized targets prostate specific antigen (PSA) and prostate-specific membrane antigen (PSMA) in the androgen-sensitive LNCaP cell line. Results in Fig. 5C indicate that compound **8c** markedly reduced the expression of AR and to a lesser extent those of PSA and PSMA. These data all together indicate that compound 8c inhibits prostate cell growth by an AMPK activation mechanism.

**Figure 6:**
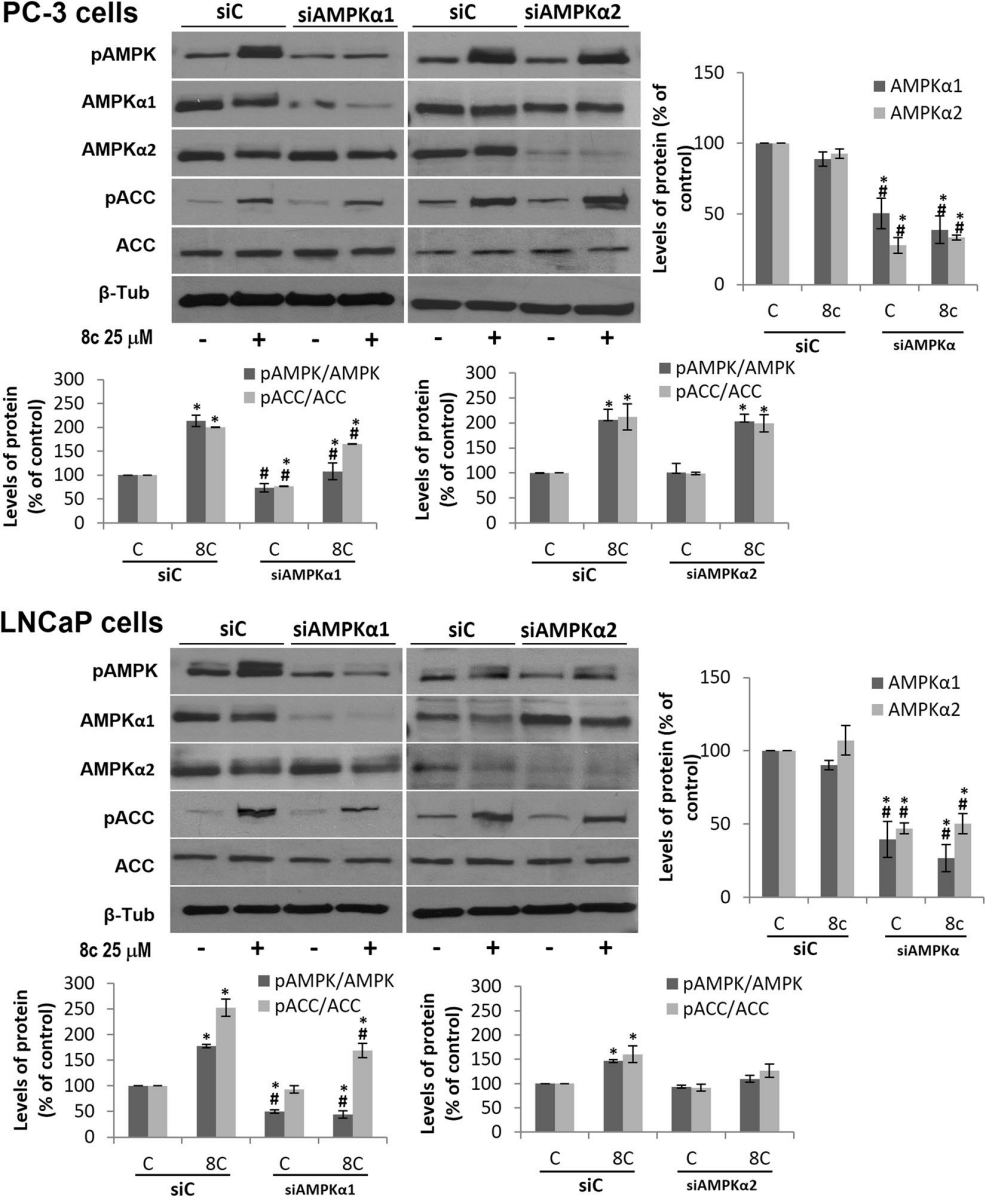
Phosphorylation of AMPK and ACC in prostate cells transfected with selective AMPKα1 or AMPKα2 siRNA. PC-3 and LNCaP cells were transfected with selective siRNA for AMPKα1 or AMPKα2 subunit isoforms and then treated with vehicle or 25 μM compound **8c** for 1 h. Levels of the phosphorylated AMPK (pAMPK), phosphorylated ACC (pACC) and their total forms were determined by Western blot. Upper panel, a representative Western blot of three different experiments. β-tubulin (β-Tub) serves as a loading control. Lower panel, densitometric analyses of bands represented as the mean of the ratio pAMPK/AMPK or pACC/ACC ± SD of three different experiments. *p <0.05 compared with the controls, ^#^p < 0.05 compared with siC by the Student’s t-test. Histogram on the right shows the rate of AMPK α subunit silencing.

AMPK is a heterotrimeric protein composed by catalytic (α) and regulatory (β and γ) subunits. In mammals, different isoforms of α, β and γ subunits can associate to produce up to 12 AMPK different combinations^42^. It is worthy to note that the most abundant form of AMPK α subunit present in PC-3 cells is α1 (AMPKα1-containig complexes are at least 10-fold more abundant than AMPKα2)^39^. To gain insight into the mechanism underlying AMPK activation by compound **8c** in prostate cells, we selectively silenced AMPKα1 or AMPKα2 in prostate cancer cells. We observed that when AMPKα1 subunit was knocked down, **8c**-induced AMPK phosphorylation was almost totally blocked, and ACC phosphorylation notably decreased (Fig. 6). In contrast, when the expression of AMPKα2 subunit was silenced compound **8c** still was able to phosphorylate AMPK and ACC at a similar level than in non-silenced (siC) cells (Fig. 6). These findings suggest that compound **8c** needs α1 subunit to exert its effect and therefore preferably activates α1 subunit-containing AMPK isoforms.

**Figure 7:**
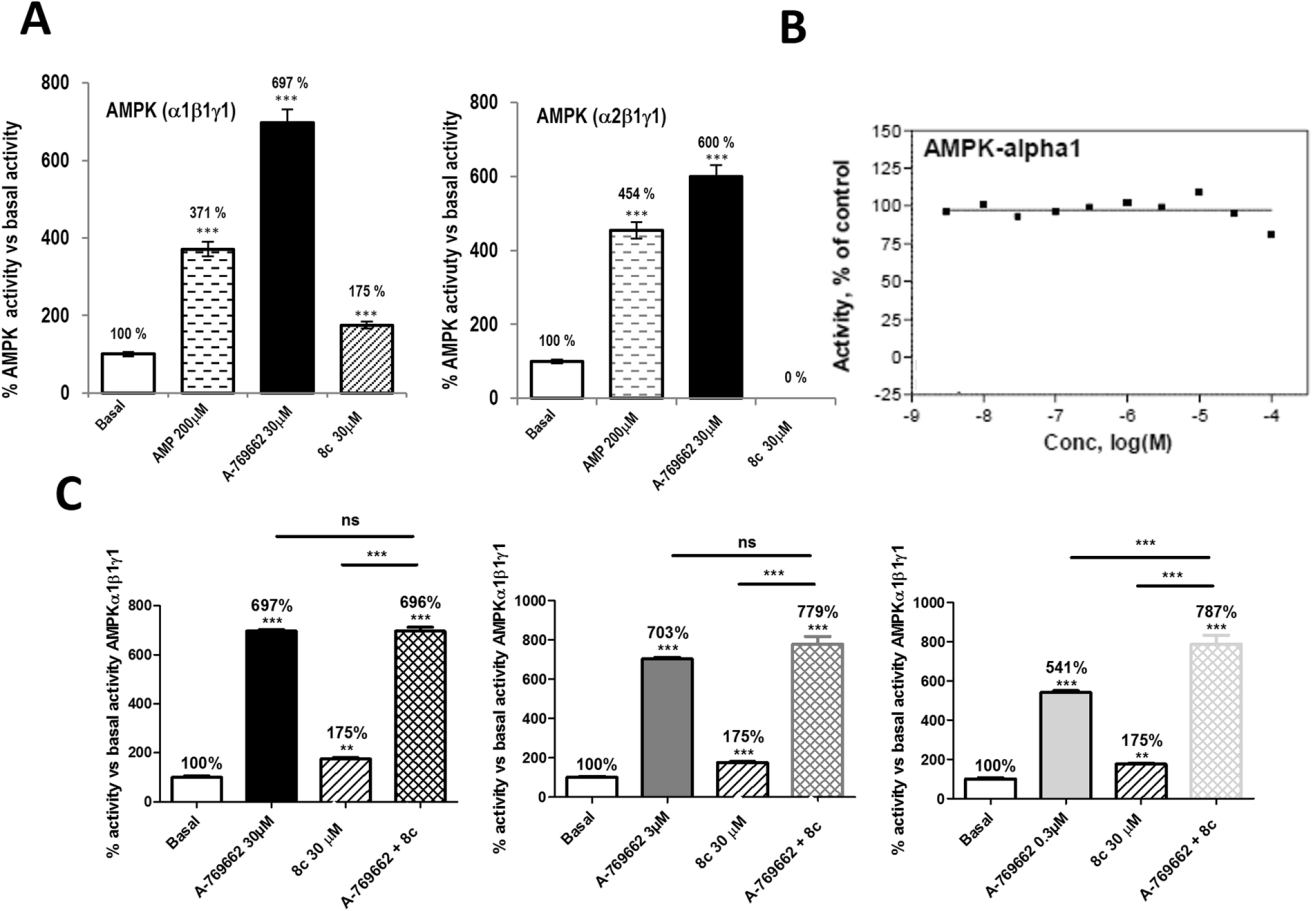
*In vitro* activity of human recombinant AMPK. (**A**) Fluorometric enzymatic assay with the human recombinant AMPK isoform α1β1γ1. This assay evaluated the ability of compound **8c** to activate AMPK isoform α1β1γ1and, in turn, phosphorylate the synthetic substrate peptide AMARAASAAALARRR. The compound was evaluated at 30 μM using AMP as a positive control and the Abbott activator A-769662 as positive reference compound. (**B**) Activity of compound **8c** on isolated AMPKα_1_ 1–550 fragment. (**C**) Co-incubation assay with the human recombinant AMPK isoform α1β1γ1. The compound **8c** was evaluated at 30 µM alone or in the presence of different concentrations of A-799662 from 0.3 µM to 30 µM. AMP was used as a positive control for the assay and A-799662 (0.3-3-30 µM) was used as a positive reference compound. Data are the mean ± S.D. of four experiments. **p < 0.01 and ***p < 0.001 compared by the Student’s t-test.

As a further proof of AMPK activation by compound **8c**, we performed *in vitro* AMPK activity assays using human recombinant α_1_β_1_γ_1_ and α_2_β_1_γ_1_ AMPK isoform. This assay evaluated the ability of the compound to activate AMPK and in turn phosphorylate the synthetic substrate peptide AMARAASAAALARRR. Figure 7A shows that compound **8c** increases AMPK α_1_β_1_γ_1_ activity in 75%, p < 0.001 whereas no activation was observed when AMPK α_2_β_1_γ_1_ isoform was used. This is an interesting finding that indicates a preference of compound **8c** for α_1_ isoform. However, it should be noted that this is a partial AMPK activation compared with AMP or the Abbott AMPK activator A-769662 which induce a higher response (Fig. 7A). We also assayed the effect of compound **8c** on a fragment of the α_1_ isoform (aa 1–550) that contains the catalytic domain. Results show that in this case there is no change in AMPK activity (Fig. 7B), inferring that compound **8c** does not bind to the catalytic site.

AMPK may be allosterically activated by AMP, which binds at up to three sites on γ subunit, or by synthetic drugs like A-769662, PF-06409577 and salicylate that directly bind and induce conformation changes in the AMPK complex enhancing protection of the activation loop against dephosphorylation^43,44^. The A-769662-binding site is located at the interface of the α-kinase domain and the β subunit and is different from the AMP binding sites^42,43,45^. Then, we analyzed AMPK α_1_β_1_γ_1_ activity when A-769662 and **8c** compounds were added together. As shown in Fig. 7C, an additive effect of **8c** and A-769662 on AMPKα_1_β_1_γ_1_ activation was only observed at a submaximal A-769662 dose (0.3 µM) when the system is not saturated. At 30 μM or 3 μM A-769662 a maximal effect on AMPKα_1_β_1_γ_1_ activation was reached and the addition of **8c** did not increase this effect. No synergy was observed at the doses used (Fig. 7C).

We then tested the selectivity of compound **8c** to a panel of 9 kinases. Among the 9 kinases analyzed, compound **8c** only showed a slight inhibitory activity on PDK1 (K_I_ = 3.1 × 10^−5^ M) and PKA K_I_ = 3.5 × 10^−5^ M) at the low micromolar range (Supplementary Table 1).

These results indicate that compound **8c** selectively activates AMPK and inhibits prostate cells proliferation and hence it was a good candidate to prove his effectiveness *in vivo*.

### In silico drug profile type evaluation of compound 8c

Previous to the *in vivo* study, we evaluated the drug-like profile of compound **8c**. One of the most common tools in the early stages of drug development is to determine the drug-like properties, because it allows making decisions based on fundamental criteria, such as permeability, oral absorption or passage of blood-brain barrier. In order to evaluate the drug-like profile of compound **8c** and to determine its potential as future drug candidate, we employed computational predictive methods. Pharmacokinetic properties of compound **8c** were predicted using the QikProp 3.5 program, integrated in Maestro (Schrödinger LLC, New York, USA). As presented in Table 2, the predicted properties of **8c** are within the ranges predicted by QikProp for 95% of known oral drugs. These data indicate that fluorinated oxindole **8c** presents an adequate druggability profile and therefore is suitable to use *in vivo*.

**Table 2:** Physicochemical descriptors of compound 8c calculated by QikProp 3.5 integrated in Maestro (Schrödinger, LLC, New York, USA).

Descriptor	Compound 8 c
*QPlogS* ^a^	−4,961
*QPPCaco* ^b^	3041,015
*QPPMDCK* ^c^	2307,16
*%Human oral absorption* (*%*)^d^	100

^a^Predicted aqueous solubility [-6.5/0.5]; ^b^Apparent Caco-2 cell permeability in nm/s [<25 poor, >500 excellent]; ^c^Apparent MDCK permeability in nm/s [<25 poor, >500 excellent] ^d^Human Oral Absorption in GI [<25% is poor]; [range of 95% of drugs].

### ***In vivo*** inhibition of PC-3 xenograft tumor growth by compound 8c

**Figure 8:**
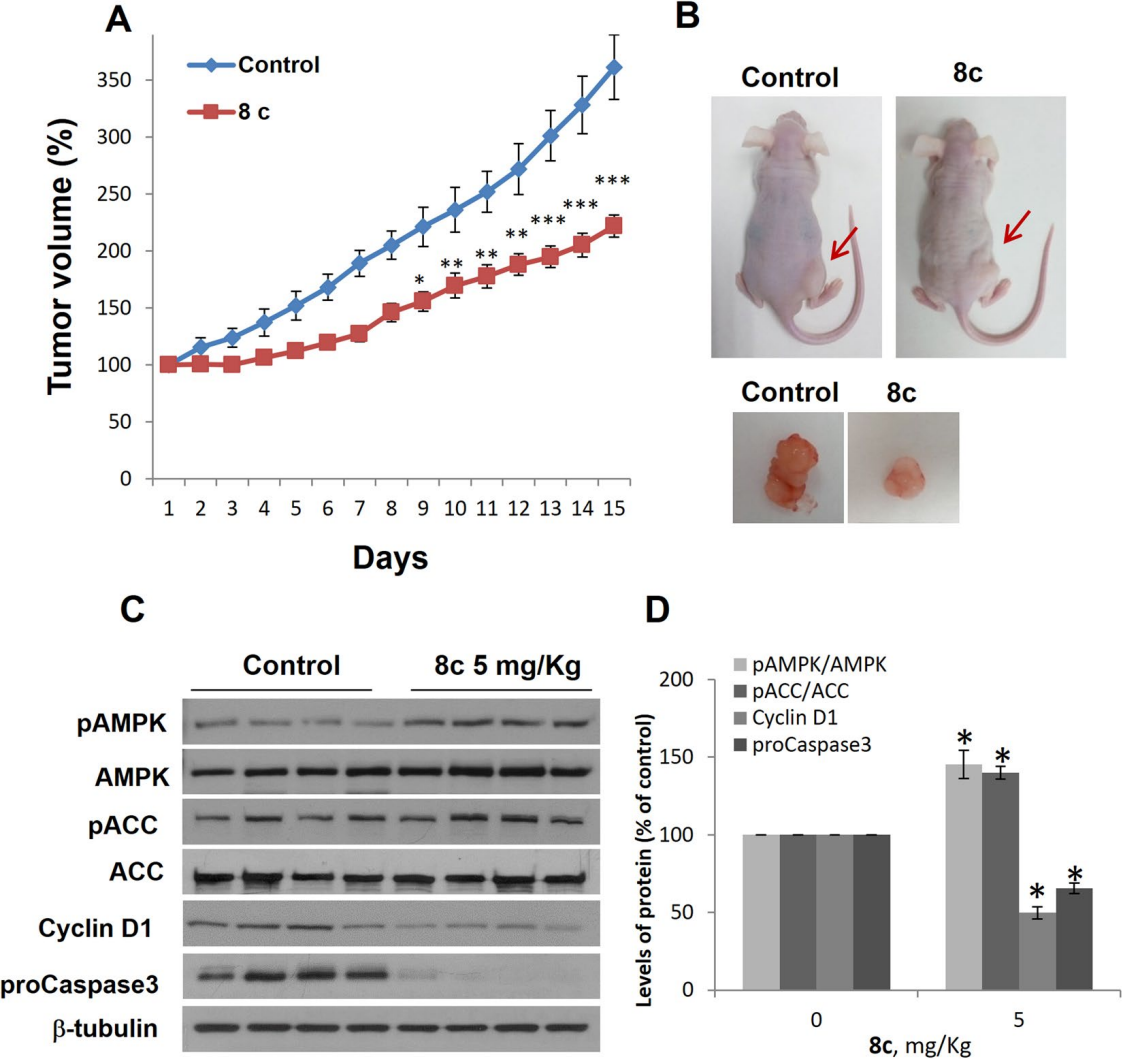
Inhibition of prostate tumor xenograft growth *in vivo* by compound 8c. (**A**) Growth curves of xenograft PC-3 tumors in mice daily treated with vehicle (Control), or 5 mg/Kg compound **8c**. Results are the mean (n = 8) ± SEM. *p < 0.05, **p < 0.01 and ***p < 0.005 compared to controls by the Student’s t test. (**B**) Pictures of representative athymic nude mice bearing PC-3 cell tumor xenografts and the dissected tumors. (**C**) levels of phospho-AMPK, phospho-ACC, Cyclin D1 and proCaspase-3, in 4 representative control mice and 4 representative **8c**-treated mice. β-tubulin serves as a loading control. (**D**) The densitometric analyses of bands represented as the mean (n = 8) ± S.D. *p < 0.05 compared with the control group by the Student’s t-test.

To validate the therapeutic value of the compound **8c** against castration-resistant prostate cancer a murine xenograft model was employed. Due to the potent effect of compound **8c** on the aggressive PC-3 cell line and in order to avoid unnecessary wasting of animals according to the 3 Rs guidelines (reduction, replacement, refinement) for the use of animals in research, we only use PC-3 cells for the *in vivo* studies. PC-3 tumors were induced in nude mice by subcutaneous injection of cells in the right flank of the mouse. When tumors reached a 70 mm^3^ volume, mice were daily treated with 5 mg/Kg of compound **8c**. In vehicle-treated mice, an increase in tumor growth volume was observed during the course of the study and at the end of experimentation (Fig. 8A). Nevertheless, treatment with compound **8c** significantly reduced tumor growth over the 15 days experimental period (Fig. 8A). The volume of the tumor at the end of the treatment was notably smaller in **8c**-treated animals than in controls (Fig. 8B). No effect on mice weight or animal welfare was observed in compound **8c**-treated mice. Serum biochemical parameters were similar between control and **8c**-treated animals and were within the reference range (Supplementary Figure 1). Liver morphology was normal either in control than in mice treated with **8c** (not shown). Consistent with our *in vitro* findings, compound **8c** promoted AMPK activation, increased ACC phosphorylation, decreased Cyclin D1 and decreased procaspase-3 levels in xenograft tumors (Fig. 8C).

These data show the novel finding that the 2-oxindole fluorinated derivative compound **8c** is effective at inhibiting the growth of prostate tumor xenografts and activating AMPK *in vivo* and accordingly has a great potential as a candidate to treat prostate cancer.

## Discussion

The cellular metabolic sensor AMPK has emerged as a key therapeutic target for many cancer types as it controls the metabolic pathways that are usually reprogrammed in cancer cells. This is especially significant in prostate cancer in which increased lipid synthesis allows the survival of cancer cells and is associated with tumor progression^5^.

In the present study we have synthesized new AMPK modulators and have selected one that exhibits potent antitumoral properties against prostate cancer cells. The small collection of fluorinated derivatives of 2-oxindole were prepared with the aim of contributing to the knowledge of some of the main structural requirements for AMPK modulation by this family of compounds. Phosphorylation assays in cellular cultures show that compounds **8a**, **8b** and **9a** are AMPK inhibitors. Nevertheless, compound **8c** shows a cellular profile suitable with an AMPK activator whereas compound **9b** slightly increases AMPK phosphorylation in PC-3 cells but not in LNCaP cells. Notably, compound **8c** is the only non-substituted compound in which a furan moiety has been introduced as substituent at C-3 position of the 2-oxindole system and is a powerful *in vivo* activator of AMPK and the best inhibitor of prostate cells growth. This suggests that this chemical feature together with the disposition of the substituent may be playing an important role in the interaction with the enzyme being important to determine the activator profile of the compound. We have identified and pharmacological characterized a new AMPK activator, compound **8c**, which exhibits potent antitumoral properties against prostate cancer cells.

The antiproliferative effect of **8c** is associated with cell cycle arrest and apoptosis. Notably, the cell cycle assays state that the compound **8c** increases subG0 cells and decreases Cyclin D1 levels. These results are in line with recent findings that underscore the importance of AMPK in the regulation of cell cycle^12,35,46^. Moreover, recent data indicate that the AMPK activator metformin inhibited proliferation of prostate cells by promoting cell cycle arrest in the G0-G1 phase, inhibiting cyclin D1 and inducing cell apoptosis^38^, which is in good agreement with our observations

According with previous research showing that AMPK induces p53 activation^47^, compound **8c** increased the phosphorylation and expression of p53 in prostate cells. In addition, **8c** modified the expression of other AMPK-dependent proteins such as p21, HIF-1A, or the androgen receptor and its targets. Our results demonstrate that compound **8c** activates AMPK in prostate cells and regulate genes involved in cell survival and growth.

The strong antiproliferative effect of compound **8c** was dependent on AMPK as suggested by the Dorsomorphin prevention and AMPK knocking down. Despite Dorsomorphin may exert unspecific AMPK-independent effects^48–50^, in our model, it inhibits AMPK activation by compound **8c** (Fig. 4A) and thus indicates that the **8c**-induced cell death was mediated, at least in part, by AMPK activation. This notion was confirmed by AMPK silencing, which blocked **8c**-induced cell death.

At cellular level, compound **8c** shows a profile suitable with an AMPK activator. This fact prompted us to investigate whether **8c** was acting on specific AMPK isoform since prostate cells primarily express α1 isoform. Knocking down of selective α isoforms and *in vitro* experiments show that compound **8c** is acting through α1-containig complexes.

There are however, several unanswered questions regarding the exact mechanism whereby **8c** activates AMPK in prostate cells. Assays with recombinant AMPK showed that compound **8c** had additive activating effect with the Abbot AMPK activator A-769662. At dose analyzed, no synergistic effect was observed, suggesting that both compounds might bind to the same site. A-769662-binding site, termed the allosteric drug and metabolite (ADaM) binding site, is located at the interface of the α-kinase domain small lobe and a surface of the carbohydrate-binding module (β-CBM) opposite to the glycogen‐binding site, as recently revealed by crystal structures of AMPK^42,43,45^. Further studies are necessary to better characterize the binding site of compound **8c**.

Finally, the antitumor activity of compound **8c** was further reinforced by the repression of xenograft tumors growth *in vivo* at a lower dose than that previously used for AMPK activators in prostate cells^12^. Moreover, in **8c**-treated tumors an increase in AMPK phosphorylation, ACC phosphorylation and a decrease in Cyclin D1 and procaspase-3was observed showing that this compound can activate AMPK also *in vivo*. In line with or results, Zadra *et al*.^12^ showed that the novel AMPK direct activator MT63–78 inhibited LNCaP prostate tumor growth *in vivo* and promoted AMPK activation in xenograft tumors.

Overall, these findings suggest that the AMPK act as a tumor suppressor in prostate cancer and therefore its activation might be a quite promising therapeutic strategy to fight against castration-resistant prostate cancer. The results shown here indicated that compound **8c** is very powerful in activating AMPK and inhibiting prostate tumor growth and accordingly is a promising candidate with therapeutic value for prostate cancer.

## Methods

### General Methods and Materials

All commercially available reagents and solvents were used without further purification. Column chromatography was performed using silica gel 60(230–400 mesh). The purity of the final compounds as determined by HPLC/MS and elemental analyses was ≥95%. Analytical HPLC/MS analysis was performed on a Waters 2695 HPLC system equipped with a photodiode array 2996 coupled to a Micromass ZQ 2000 mass spectrometer (ESI-MS) using a reversed-phase SunFire column (C-18, 4.6 × 50 mm, 3.5 μm) and a 5 min gradient of solvents A (MeCN/0.08% formic acid) and B (H_2_O/1% formic acid) visualizing at λ = 254 nm. Elemental analyses of all synthesized compounds were performed using a LECO CHNS-932 apparatus. Deviations of the elemental analysis results from the calculated values are within ± 0.4%. ^1^H and ^13^C NMR spectra were recorded on a Bruker 300 (300 and 75 MHz) at 25 °C. Samples were prepared as solutions in deuterated solvent and referenced to an internal non-deuterated solvent peak. Chemical shifts are expressed in parts per million (δ) downfield of tetramethylsilane. Melting points were determined on an MP70 Mettler Toledo apparatus.

### General procedure for 3-methylen-2-oxindoles (6a-c)

To a solution of 2-oxindole (1 eq.) in MeOH (25 mg/mL), the corresponding aldehyde (1 eq.) was added. The reaction mixture was heated at reflux for 3 h. After cooling at 0 °C, the product was isolated by filtration.

#### (E, Z)-3-((1H-pyrrol-2-yl)methylene)indolin-2-one (**6a**)

Following the general procedure for 3-methylen-2-oxindoles, reaction of pyrrole-2-carboxaldehyde (707.4 mg, 7.44 mmol) and 2-oxindole (990 mg, 7.44 mmol) yield 502 mg (64%) of **6a** as yellow solid: mp 215–217 °C (bibl. 215–220 °C)^51^; ^1^H NMR (CDCl_3_) δ 13.18 (s, 1 H, NH), 8.07 (s, 1 H), 7.45–7.31 (m, 2 H), 7.14–7.07 (m, 2 H), 6.99 (t, J = 7.5 Hz, 2 H), 6.83 (d, J = 7.7 Hz, 1 H), 6.70 (s, 1 H), 6.32 (s, 1 H); HPLC t_R_ = 4.74 min (99%); MS (ES^+^) m/z 210.9 [M + H]^+^.

#### (E, Z)-5-chloro-3-[4-(pyrrolidin-1-yl)benzyliden]indolin-2-one (**6b**)

Following the general procedure for 3-methylen-2-oxindoles, reaction of 4-(1-pyrrolidin)benzaldehyde (313.68 mg, 1.79 mmol) and 5-chloro-2-oxindole (300 mg, 1.79 mmol) yield 366 mg (37%) of **6b** as yellow oil: ^1^H NMR (DMSO-d_6_) δ 11.14 (s, 1 H, NH), 9.64 (s, 2 H), 7.72–7.52 (m, 4 H), 6.93 (d, J = 8.4 Hz, 2 H), 6.63 (d, J = 8.5 Hz, 2 H), 3.41–3.33 (m, 4 H, N-CH2), 1.98 (t, J = 6.7 Hz, 4 H, N-CH2-CH2); HPLC: t_R_ = 3.38 min (99.9%); MS (ES^+^) m/z 323.0 [M-2H]^+^.

#### (E, Z)-3-((5-phenylfuran-2-yl)methylene)indolin-2-one (**6c**)

Following the general procedure for 3-methylen-2-oxindoles, reaction of 5-phenyl-2-furaldehyde (640.34 mg, 3.72 mmol) and 2-oxindole (495 mg, 3.72 mmol) yield 445 mg (46%) of **6c** as orange solid. mp 230 °C; ^1^H NMR (DMSO-d_6_) δ 10.59 (s, 1 H), 8.49 (d, J = 7.8 Hz, 1 H), 7.93 (d, J = 7.7 Hz, 2 H), 7.57 (t, J = 7.7 Hz, 3 H), 7.50–7.23 (m, 6 H), 7.12 (t, J = 7.6 Hz, 1 H), 6.92 (d, J = 7.7 Hz, 1 H); HPLC t_R_ = 5.32 min (99%); MS (ES^+^) m/z 288.0 [M + H]^+^.

### General procedure for 3-(arylmethyl)-2-oxindoles (7a-7c)

To a solution of the corresponding 3-methylen-2-oxindole (1 eq.) in MeOH (10 mg/mL), NaBH_4_ (5 eq.) was added during 30 min slowly. The reaction mixture was stirred at rt overnight. The resulting mixture was then quenched by water (25 mL), which was extracted with EtOAc (3 × 30 mL). The combined extracts were dried (Na_2_SO_4_) and concentrated under reduced pressure, and the crude was purified by flash column chromatography using mixtures of ethyl acetate:hexane (1:1) to give compounds **7a**-**7c**.

#### (±) (R, S)-3-((1H-pyrrol-2-yl)methyl)indolin-2-one (**7a**)

Following the general procedure for 3-(arylmethyl)-2-oxindoles, reaction of **6a** (400 mg, 1.90 mmol) and NaBH_4_ (366,96 mg, 9.51 mmol) yield 110 mg (27%) of **7a** as yellow solid: mp 173–175 °C; ^1^H NMR (CDCl_3_) δ 9.21 (s, 1 H, NH), 7.90 (s, 1 H, NH), 7.30–7.03 (m, 4 H, H-Ar), 6.89 (d, J = 7.6 Hz, 1 H, H-Ar), 6.74 (m, 1 H, H-Ar), 6.12 (m, 1 H, H-Ar), 5.97 (m, 1 H, H3), 3.76 (t, J = 6.3 Hz, 1 H, CH_2_), 3.31–3.21 (m, 1 H, CH_2_); ^13^C NMR (DMSO-d_6_) δ 178.58, 140.49, 131.15, 128.12, 127.00, 124.62, 123.51, 117.59, 110.94, 108.44, 103.92, 40.30, 28.19; HPLC t_R_ = 3.73 min (98%); MS (ES^+^) m/z 213.0 [M + H]^+^.

#### (±) (R, S)- 5-chloro-3-(4-(pyrrolidin-1-yl)benzyl)indolin-2-one (**7b**)

Following the general procedure for 3-(arylmethyl)-2-oxindoles, reaction of **6b** (150 mg, 0.46 mmol) and NaBH_4_ (91 mg, 2.31 mmol) yield 75 mg (50%) of **7b** as orange oil: ^1^H NMR (DMSO-d_6_) δ 8.04 (d, J = 1.2 Hz, 1 H, H-Ar), 7.43–7.37 (m, 2 H, H-Ar), 7.31–7.22 (m, 2 H, H-Ar), 6.64–6.58 (m, 2 H, H-Ar), 4.24–4.17 (m, 1 H), 3.88 (dd, J = 9.4, 7.6 Hz, 2 H), 3.67–3.53 (m, 2 H), 3.48–3.21 (m, 2 H), 3.09–2.94 (m, 1 H), 2.13 (m, 2 H), 1.79–1.30 (m, 2 H); 13 C NMR (DMSO-d_6_) δ 178.25, 149.90, 139.61, 132.21, 130.19, 129.75, 129.47, 129.30, 124.55, 115.77, 112.16, 49.15, 47.32, 34.51, 25.39; HPLC t_R_ = 3.40 min (99.9%); MS (ES^+^) m/z 323.2 [M-2H]^+^.

#### (±) (R, S)-3-((5-phenylfuran-2-yl)methyl)indolin-2-one (**7c**)

Following the general procedure for 3-(arylmethyl)-2-oxindoles, reaction of **6c** (500 mg, 1.74 mmol) and NaBH_4_ (335.64 mg, 8.70 mmol) yield 226 mg (45%) of **7c** as pale orange solid: mp 201–203 °C; 1 H NMR (DMSO-d_6_) δ 10.43 (s, 1 H, NH), 7.53 (d, J = 7.1 Hz, 2 H, CH-C-furyl), 7.36 (t, J = 7.7 Hz, 2 H, CH-CH-C-furyl), 7.23 (t, J = 7.3 Hz, 1 H, H-Ar), 7.14 (t, J = 7.7 Hz, 1 H, H-Ar), 7.02 (d, J = 7.4 Hz, 1 H, H-Ar), 6.89 (t, J = 6.9 Hz, 1 H, H-Ar), 6.79 (d, J = 7.7 Hz, 1 H, H-Ar), 6.75 (d, J = 3.2 Hz, 1 H, H-Ar), 6.54 (m, 1 H, H-Ar), 6.11 (d, J = 3.3 Hz, 1 H, H3), 3.83 (dd, J = 7.3, 4.9 Hz, 1 H, CH_2_), 3.10 (dd, J = 15.4, 7.4 Hz, 1 H, CH^2^); ^13^C NMR (DMSO-d_6_) δ 178.19, 152.43, 151.97, 143.09, 130.70, 129.21, 129.10, 128.10, 127.38, 124.45, 123.35, 121.46, 109.53, 109.47, 106.73, 44.61, 28.37; HPLC t_R_ = 4.92 min (98%); MS (ES^+^) m/z 290.0 [M]^+^.

### General procedures for 3-(arylmethyl)-3-fluoro-2-oxindoles (8a-8c)

To a solution of the corresponding 3-(arylmethyl)-2-oxindole (1 eq.) in MeOH (5 mg/mL) cooled at 0 °C, Selectfluor (2 eq.) was added slowly. The reaction mixture was stirred at 0 °C for 7 h, concentrated and the crude was purified by flash column chromatography using mixtures of ethyl acetate:hexane (1:1) to give compounds **8a**-**8c**.

#### (±) (R, S)- 3-((1H-pyrrol-2-yl)methyl)-3-fluoroindolin-2-one (**8a**)

Following the general procedure for 3-(arylmethyl)-3-fluoro-2-oxindoles, reaction of **7a** (100 mg, 0.708 mmol) and Selectfluor (501.28 mg, 1.415 mmol) yield 20 mg (18%) of **8a** as yellow solid: mp 143–146 °C; ^1^H NMR (CDCl_3_) δ 8.21 (s, 1 H, NH), 7.81 (dd, J = 5.6, 3.4 Hz, 1 H, H4), 7.37 (m, 2 H, H5 + H6), 7.25 (dd, J = 5.6, 3.5 Hz, 1 H, H7), 6.78 (dd, J = 7.5, 1.5 Hz, 1 H, H5′), 6.29 (t, J = 7.5 Hz, 1 H, H4′), 6.02 (dd, J = 7.5, 1.5 Hz, 1 H, H3′), 3.86 (dd, J = 25.2, 12.4 Hz, 1 H, CH_2_), 3.12 (dd, J = 25.2, 12.4 Hz, 1 H, CH_2_); 13 C NMR (DMSO-d_6_) δ 172.14 (d, J = 34.2 Hz, C2), 141.68, 133.22, 126.94, 124.77, 122.10, 120.49, 120.23, 117.41, 109.97, 107.44, 94.76 (d, J = 268.3 Hz, C3), 28.76 (d, J = 28.0 Hz, CH_2_); MS (ES^+^) 231.8 [M + H]^+^. Calcd for C_13_H_11_FN_2_O: C 67.82, H 4.82, N 12.17. Found: C 67.76, H 4.71, N 12.25.

#### (±) (R, S)-5-chloro-3-fluoro-3-(4-(pyrrolidin-1-yl)benzyl)indolin-2-one (**8b**)

Following the general procedure for 3-(arylmethyl)-3-fluoro-2-oxindoles, reaction of **7b** (50 mg, 0.15 mmol) and Selectfluor (108.40 mg, 0.3 mmol) yield 27 mg (53%) of **8b** as red oil: ^1^H NMR (DMSO-d6) δ 9.64 (s, 1 H), 7.65 (d, J = 8.8 Hz, 2 H, H3′), 7.31–7.05 (m, 2 H, H6 + H7), 6.74 (d, J = 8.0 Hz, 1 H, H4), 6.50 (d, J = 8.6 Hz, 2 H, H4′), 3.46 (m, 2 H, CH_2_), 3.32 (t, J = 6.7 Hz, 4 H, H7′), 2.03–1.92 (m, 4 H, H8′); ^13^C NMR (DMSO-d_6_) δ 172.25 (d, J = 32.4 Hz, C2), 150.28, 141.22, 130.85, 129.57, 129.27, 129.11, 124.02, 121.55, 116.43, 111.07, 94.34 (d, J = 268.1 Hz, C3), 49.05 (N-CH_2_), 39.97 (d, J = 27.7 Hz, CH2), 25.36 (N-CH_2_-CH_2_); HPLC t_R_ = 0.55 min (99.9%); MS (ES^+^) m/z 343.3 [M-H]^+^. Calcd for C_19_H_18_ClFN_2_O: C 66.18, H 5.26, N 8.12, Cl 10.28. Found: C 66.42, H 4.96, N 7.89, Cl 10.31.

#### (±) (R, S)-3-fluoro-3-((5-phenylfuran-2-yl)methyl)indolin-2-one (**8c**)

Following the general procedure for 3-(arylmethyl)-3-fluoro-2-oxindoles, reaction of **7c** (200 mg, 0.70 mmol) and Selectfluor (489.77 mg, 1.40 mmol) yield 40 mg (18%) of **8c** as yellow solid: mp 215–218 °C; ^1^H NMR (DMSO-d_6_) δ 7.85 (dd, J = 7.4, 1.5 Hz, 1 H), 7.88–7.74 (m, 2 H), 7.59 (dt, J = 7.5, 1.5 Hz, 1 H), 7.55–7.39 (m, 4 H), 7.27 (dd, J = 7.5, 1.6 Hz, 1 H), 7.00 (d, J = 7.3 Hz, 1 H), 6.56 (d, J = 7.5 Hz, 1 H), 3.77 (dd, J = 25.1, 12.4 Hz, 1 H, CH2), 3.42 (dd, J = 25.2, 12.4 Hz, 1 H, CH_2_); ^13^C NMR (DMSO-d_6_) δ 172.15 (d, J = 32.4 Hz, C2), 149.42, 144.16, 141.65, 129.34, 128.20, 127.84, 126.94, 124.77, 124.75, 122.10, 120.49, 120.23, 110.46, 109.97, 109.29, 93.60 (d, J = 268.1 Hz, C3), 33.37 (d, J = 26.7 Hz, CH2); HPLC t_R_ = 2.56 min (99.9%). MS (ES^+^) m/z 306.8 [M]^+^. Calcd for C_19_H_14_FNO_2_: C 67.82, H 4.82, N 4.56. Found: C 67.76, H 4.59, N 4.16.

### General procedures for 3-(arylmethyl)-1-benzyl-3-fluoro-2-oxindoles (9a-9b)

To a stirred solution of the corresponding 3-(arylmethyl)-3-fluoro-2-oxindole (1 eq.) in anhydrous DMF (12 mg/mL), NaH (1.1 eq.) was added at rt. After 10 min., benzyl bromide (1 eq.) was added and the reaction mixture was stirred for 15 min at rt. Finally, brine (10 mL) was added under ice-cooling and the resulting precipitate was filtered off and purified by flash column chromatography using mixtures of ethyl acetate:hexane (3:1) to give compounds **9a**-**9b**.

#### (±) (R, S)-3-((1H-pyrrol-2-yl)methyl)-1-benzyl-3-fluoroindolin-2-one (**9a**)

Following the general procedure for 3-(arylmethyl)-1-benzyl-3-fluoro-2-oxindoles, reaction of **8a** (50 mg, 0.156 mmol), NaH (4.12 mg, 0.172 mmol) and benzyl bromide (26.68 mg, 0.156 mmol) yield 10 mg (15%) of **9a** as beige solid: mp 121–124 °C; ^1^H NMR (DMSO-d_6_) δ 7.80 (m, 1 H, H4), 7.38–7.11 (m, 9 H), 6.28 (d, J = 7.5 Hz, 1 H, H5″), 5.99 (t, J = 7.5 Hz, 1 H, H4″), 5.81 (d, J = 7.5 Hz, 1 H, H3″), 5.53–5.47 (m, 1 H, CH_2_-Ph), 5.33–5.26 (m, 1 H, CH_2_-Ph), 3.65 (dd, J = 25.2, 12.3 Hz, 1 H, CH_2_-C-F), 3.42 (dd, J = 25.1, 12.4 Hz, 1 H, CH_2_-C-F); ^13^C NMR (DMSO-d_6_) δ 178.9 (d, J = 29.8 Hz, C2), 140.7, 139.3, 130.1, 129.5, 129.0, 127.6, 126.6, 124.4, 120.5, 117.6, 116.0, 108.0, 107.4, 106.1, 90.0 (d, J = 269.0 Hz, C3), 49.2 (C1′), 22.8 (d, J = 27.6 Hz, FC-CH_2_); HPLC t_R_ = 3.39 min (99.9%); MS (ES^+^) m/z 323.1 [M + 2 H]^+^. Calcd for C_20_H_17_FN_2_O: C 74.98, H 5.35, N 8.74. Found: C 75.25, H 4.93, N 8.81.

#### (±) (R, S)-1-benzyl-5-chloro-3-fluoro-3-(4-(pyrrolidin-1-yl)benzyl)indolin-2-one (**9b**)

Following the general procedure for 3-(arylmethyl)-1-benzyl-3-fluoro-2-oxindoles, reaction of **8b** (50 mg, 0.145 mmol), NaH (3.83 mg, 0.160 mmol) and benzyl bromide (24.8 mg, 0.145 mmol) yield 30 mg (47%) of **9b** as orange oil: ^1^H NMR (CDCl_3_) δ 7.61 (dd, J = 7.5, 2.0 Hz, 1 H, H6), 7.51 (d, J = 7.5 Hz, 1 H, H7), 7.36 (d, J = 1.9 Hz, 1 H, H4), 7.29–7.20 (m, 5 H), 7.08–7.02 (m, 2 H), 6.61 (d, J = 7.5 Hz, 2 H, H3′′), 5.73 (m, 1 H, CH_2_-Ph), 5.24 (m, 1 H, CH_2_-Ph), 3.89 (m, 2 H, H6′′), 3.70–3.56 (m, 3 H), 2.98 (m, 1 H, CH_2_-CF), 2.20–2.11 (m, 2 H, H7′′), 1.73–1.61 (m, 2 H, H7′′); ^13^C NMR (DMSO-d6) δ 173.21 (d, J = 32.4 Hz, C2), 150.60, 143.23, 136.82, 131.51, 131.07, 130.03, 128.57, 127.66, 127.55, 127.51, 124.45, 124.20, 121.31, 116.60, 110.10, 95.68 (d, J = 268.1 Hz, C3), 49.15 (N-CH_2_), 45.98 (CH_2_-Ph), 39.94 (d, J = 26.7 Hz, F-C-CH_2_), 25.39 (N-CH_2_-CH_2_); HPLC t_R_ = 4.69 min (98%); MS (ES^+^) m/z 432.2 [M-2H]^+^. Calcd for C_21_H_17_FN_2_O: C 75.89, H 5.16, N 8.43. Found: C 76.12, H 5.43, N 8.66.

### *In vitro* AMPK enzymatic assay

The effect of compounds in the *in vitro* human recombinant AMPK activity assay was measured by monitoring phosphorylation of the “AMARA” peptide using Delfia technology. Briefly, AMPK enzyme activities were carried out in 96 well microtiter plates (containing 50 mM Hepes buffer, pH 7.4 with 125 µM ATP and 19 mM MgCl2) in the presence of the human recombinant AMPKα_2_β_1_γ_1_ or AMPKα_1_β_1_γ_1_ proteins (Invitrogen, Carlsbad, CA, USA), 1 mM synthetic peptide substrate (AMARAASAAALARRR, the “AMARA” peptide), and the compounds. Final concentration of DMSO was 1%. Reactions were initiated by the addition of AMPK (0.1U). Following mixing, the plates were incubated for 30 min at room temperature. Enzyme activity was assayed by using an anti-phospho-serine antibody to measure the quantity of phosphate incorporated into the AMARAA peptide.

### Protein Kinase Assay

A radiometric protein kinase assay (33PanQinase® Activity Assay) was used for measuring the kinase activity of the 10 protein kinases according to the manufacturer’s (Biochemical Screening Systems, ProQinase GmbH, Freiburg, Germany). IC50 values were measured by testing 10 concentrations of the compounds in the range from 1 × 10–04 M to 3 × 10–09 M in duplicate in each kinase assay. The assay for all protein kinases contained 70 mM HEPES-NaOH pH 7.5, 3 mM MgCl2, 3 mM MnCl2, 3 μM Na-orthovanadate, 1.2 mM DTT, ATP (variable amounts, corresponding to the apparent ATP-Km of the respective kinase), [γ-^33^P]-ATP (9 × 10^05^ cpm per well), the target human recombinant kinase (ProQinase GMBH^®^, Freiburg, Germany) and substrate. The reaction cocktails were incubated at 30 °C for 60 minutes. The reaction was stopped with 50 μl of 2% (v/v) H_3_PO_4_, plates were aspirated and washed two times with 200 μl 0.9% (w/v) NaCl. Kinase activity dependent transfer of ^33^Pi (counting of “cpm”) was determined with a microplate scintillation counter (Microbeta, Wallac). All assays were performed with a BeckmanCoulter Biomek 2000/SL robotic system.

### Cell culture

Human prostate cancer epithelial PC-3, DU145 and LNCaP and human prostate normal RWPE-1 cells were purchased from American Type Culture Collection (ATCC CRL-1435 CRL-1740 and CRL 11609 respectively) (Rockville, MD, USA). The human prostate normal PNT2 cell line was purchased to ECACC (European Collection of Cell cultures, Salisbury, SP4 0JG, UK). Cells were routinely grown in RPMI 1640 medium supplemented with 100 IU/ml penicillin G sodium, 100 µg/ml streptomycin sulfate, 0.25 µg/ml amphotericin B (Invitrogen, Paisley, UK) and 10% fetal calf serum. Cells were used at passages between 10–20. For treatment experiments, cells were plated and grown over night, the medium was then replaced with serum-free RPMI 1640 for 24 hours and then incubated with different doses of the compounds for the indicated times.

### Cell viability

Cell viability was determined by 3-(4,5-dimethylthiazol-2-yl)-2,5-diphenyltetrazolium bromide (MTT) (Sigma-Aldrich) method. In brief, a total of 5000 cells/well were seeded into 12-well plate in a final volume of 1 mL. After treatments, 20 µl MTT solution (5 mg/ml in PBS) was added to the medium and cells were incubated at 37 °C for 4 h. Then, the supernatant was discarded and isopropanol was added to dissolve the formazan crystals. Treatments were carried out in triplicate. The optical density in each well was evaluated by measurement of absorbance at 490 and 650 nm using a microplate reader (ELX 800 Bio-Tek Intruments, INC). IC50 values were calculated using GraphPad Prism vs 6.0 (GraphPad Software, Inc, La Jolla, CA, USA). Cell viability was also determined by counting viable and dead cells by Trypan blue staining. Trypan blue positive and negative cells were counted using a Countess^TM^ automated cell counter (Invitrogen, Carlsbad, CA, USA). Results were expressed in relation to total number of cells counted.

### Cell Cycle analysis

Flow cytometry was used to detect the distribution of cell cycle. After being cultivated with the treatment, 10^5^ cells in 35 mm culture dish were harvested in 0.35% trypsin, collected and fixed with 70% cold ethanol at 4 °C for 1 h. Then, cells were centrifuged at 1500 g for 5 min and incubated in 0.5 ml PBS containing 0.1 mg/ml RNase for 30 min at 37 °C. DNA staining was performed adding 5 μl Propidium Iodide (Invitrogen, Eugene, Oregon, USA). Data acquisition and analysis were performed in a FACSCalibur flow cytometry system (BD Biosciences, San Jose, CA, USA) using Cyflogic software V1.2.1 (Perttu Terho, Mika Korkeamaki, CyFlo Ltd, Turku, FINLAND). A total of 5 × 10^3^ events were collected for each sample.

### Western blotting

Cells were lysed in Lysis buffer (50 mM Tris pH 7.4, 0.8 M NaCl, 5 mM MgCl2, 0.1% Triton X-100) containing Protease Inhibitor and Phosphatase inhibitor Cocktails (Roche, Basel, Switzerland). Protein concentrations were measured by BioRad™ protein assay kit (Richmond, CA, USA). Equal amount of protein (20 μg) from each sample was separated on 8% SDS–PAGE gels and transferred to PVDF membranes (BioRad). The membranes were blocked with blocking buffer (1× TBS, 0.1% Tween 20 with 5% w/v fat-free dry milk), washed and incubated with primary antibodies at 4 °C for 12 h. The membranes were subsequently washed with TBS-T (1× TBS and 0.1% Tween 20) and incubated with horseradish peroxidase-conjugated anti-mouse or anti-rabbit IgG antibodies (Sigma, St Louis, MO, USA) at room temperature for 1 h. The immune complex was visualized with an ECL system (Cell Signaling Technology). Films were analyzed by densitometry. The anti-pAMPKα1-thr172, pACC-ser79, pP53, P21, pRaptor and the antibodies against the corresponding total forms were obtained from Cell Signaling Technology (Danvers, MA, USA). The anti-caspase 3 and anti-procaspase 3 were from Santa Cruz Biotechnology (Dallas, Texas, USA). The anti-HIF1A was from Novus Biologicals LLC (Littleton, CO, USA). The anti-Cyclin D1 antibody was obtained from ThermoFisher (Alcobendas, Madrid, Spain). All the other chemicals were obtained from Sigma (St. Louis, MO, USA).

### Knockdown of AMPK α1 AMPK α2

For AMPK α1 and α2 subunits knocking down, LNCaP and PC3 cells were transfected with 5 nM of selective small interfering siRNAs for AMPK α1 or α2 subunits or a negative control siRNA (Ambion-Life Technologies, Carlsbad, CA, USA) using lipofectamine RNAi Max (Life Technologies, Spain) and following the manufacturer’s instructions. Control of proteins silencing was carried out by Western blot using AMPK α1 and AMPK α2 specific antibodies (#2795 and #2757, respectively, Cell Signaling Technology, Danvers, MA, USA).

### *In Silico* ADME Calculations

A set of 34 physicochemical descriptors were computed using QikProp version 3.5 integrated in Schrödinger Molecular Modeling Suite (Schrödinger Release 2015-4, Schrödinger, LLC, New York). The QikProp descriptors are shown in Table 2. The 3D structures used in the calculation of QikProp descriptors were generated using Maestro (version 10.4,) and energy minimizations were carried out using Macromodel (version 9.9). Local minimum energy structures of each compound were used as input for ADME studies with QikProp.

### Experimental animal

The *in vivo* experiments were performed in accordance with EU Directive 2010/63/EU and Institutional guidelines as defined by Institutional Experimental Animal Investigation Ethical Committee of Alcalá University and in compliance with Spanish current legislation (Royal Decree 53/2013). Protocols used were approved by Institutional Animal Care and Use Committee from Comunidad de Madrid (Ref. PROEX241/15).

Male immunodeficient nude-Foxn1 (nu/nu) mice (Envigo RMS, Barcelona, Spain), 4 to 6 week old, were used for all xenograft studies. Mice were housed under specific pathogen-free conditions in a 12-hour light-dark cycle at 21–23 °C and 40–60% humidity with access to food pellets and tap water *ad libitum*. Tumors were induced by s.c. flank injection of 5 × 10^6^ (100 μL) PC-3 cells in PBS + 0.5% BSA. When tumors were palpable, the animals were assigned randomly to two groups (*n* = 8) and subcutaneously injected with compound **8c** (5 mg/kg/d, which considering an average animal weight of 28 g correspond to 128 μM), or vehicle (5% ethanol in PBS) for 15 days. Tumors were measured with external calipers and volume was calculated by the modified ellipsoidal formula as volume = length × (width)^2^ × 0.52 mm^3^ ^52,53^. At the end of the study, the mice were sacrificed by placing them in a CO2 gas-filled chamber, and the excised tumors were recovered and homogenized in lysis buffer for protein quantification and Western blotting.

### Serum Clinical parameters determination

The 9 analytes assayed constitute the routine biochemistry profile. The analyses were performed with an ADVIA 1800 Chemistry System (Siemens Healthcare GmbH, Erlangen, Germany).

### Statistical analysis

Statistical analysis of the data was performed by one-way ANOVA test followed by Fisher’s least significant difference (LSD) or Tukey multiple comparison test, using the 95% confidence interval. When indicated, the Student’s t test was performed for comparison of AMPK activation between **8c**-treated and control groups. Statistical was performed using Microsoft Office Excel 2013. Significance levels were defined as P < 0.05 (*,#) and P < 0.01 (**,##). All graphs were drawn using Origin software.

### Data Availability

The datasets generated during and/or analysed during the current study are available from the corresponding author on reasonable request.

## Electronic supplementary material

Dataset 1
