# Supplementary material for: Identification of a novel 2-oxindole fluorinated derivative as in vivo antitumor agent for prostate cancer acting via AMPK activation
Source: Sci Rep. 2018 Mar 12;8:4370. doi: 10.1038/s41598-018-22690-2 (PMC5847527; doi:10.1038/s41598-018-22690-2)
Supplement: Supplementary file 1 — Dataset 1 [file 41598_2018_22690_MOESM1_ESM.doc]

**Identification of a novel 2-oxindole fluorinated derivative as in vivo antitumor agent for prostate cancer acting via AMPK activation**

**Alicia Bort^1^*, Sergio Quesada^2^*, Ágata Ramos-Torres^1^, Marta Gargantilla^2^, Eva María Priego^2^, Sophie Raynal^3^, Franck Lepifre^3^, Jose M Gasalla^1,4^ Nieves Rodriguez-Henche^1^, Ana Castro^2#^, Inés Díaz-Laviada^1,5#^**

| **Kinase** | **K_I_** |
| --- | --- |
| AKT1 aa1-480 | > 1x10^-4^ |
| CAMKK1 | > 1x10^-4^ |
| CAMKK2 | > 1x10^-4^ |
| CDK1/CycB1 | > 1x10^-4^ |
| CDK2/CycD1 | > 1x10^4^ |
| ERK1 | > 1x10^-4^ |
| PDK1 | 3.1x10^-5^ |
| PKA | 3.5x10^-5^ |
| PKC-alpha | > 1x10^-4^ |
|  |  |

**Supplementry Table 1.** **Inhibitory profile of compound 8c on different protein kinases.** K_I_ values were measured by testing 10 concentrations of the compound in the range from 1 x 10^-4^ M to 3 x 10^-9^ M in duplicate in each kinase assay.


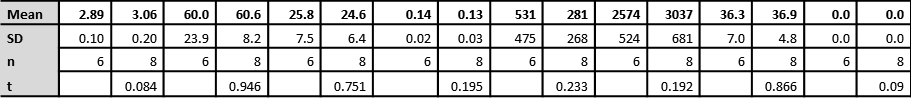

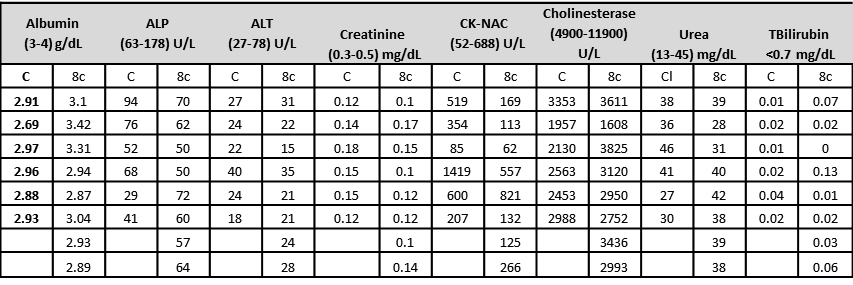


**Serum Clinical parameters of control and treated mice**

**Supplementary Table 2. Serum Clinical parameters of PC-3 xenograft mice.**. Mice blood was extracted at the end of the treatment by cardiac puncture and sera obtained by centrifugation. Biochemical parameters were determined in the sera of animals by Albumin, alkaline phosphatase (ALP), alanine aminotransferase (ALT), creatinine, creatine kinase (CK-NAC), cholinesterase, urea and total bilirubin (TBilirubin) were analyzed in the serum of control (n=6) and **8c**-treated (n=8) mice. The serum of two control animals was insufficient for ADVIA 1800 analyzer. Reference ranges and units are shown on the top.

**pACC**

**ACC**

**pAMPK**

**AMPK**

**β-Tub**

**8c 25 μM**

**PC-3 cells**


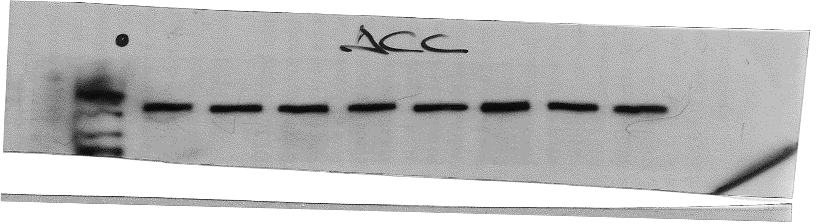

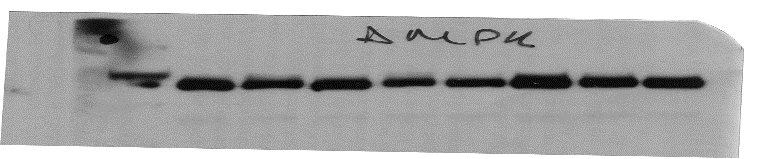

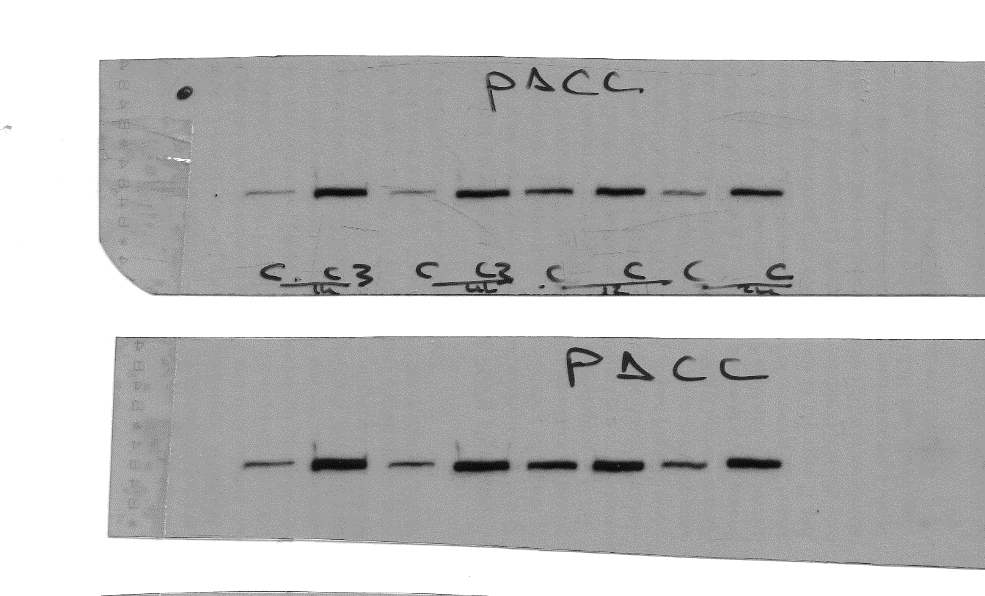

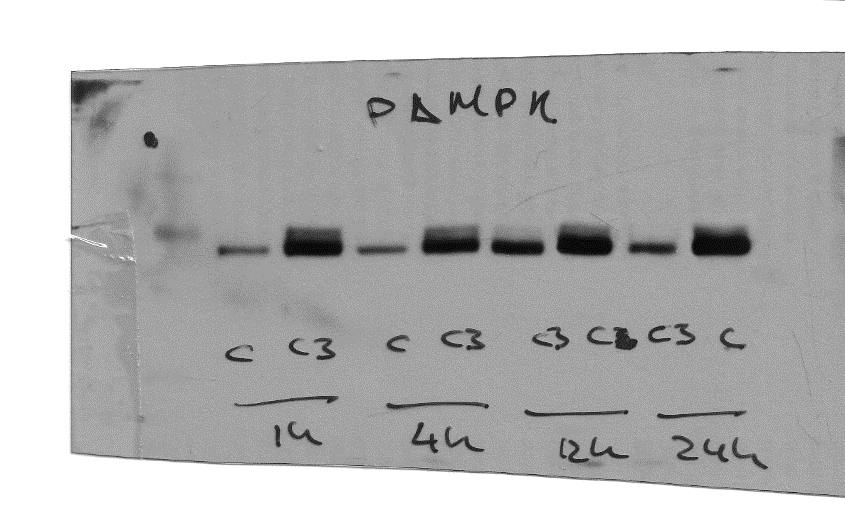


**+**

**-**

**+**

**-**

**+**

**-**

**+**

**-**

**1 h**

**4 h**

**12 h**

**24 h**


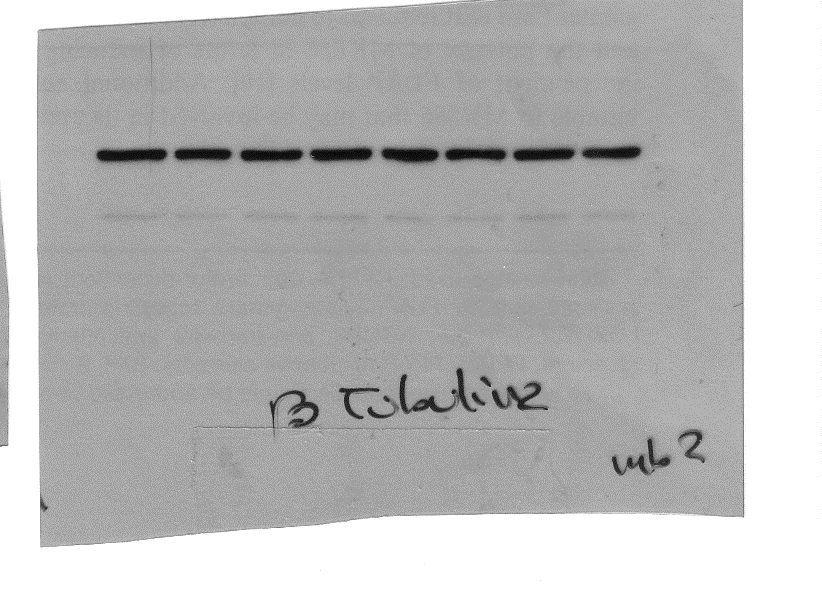


**Supplementary Figure 1. Time course of AMPK activation in 8c-treated prostate cancer cells**. PC-3 cells were treated with compound **8c** for 1, 4, 12 and 24 hours and levels of the phosphorylated AMPK (pAMPK) and phosphorylated ACC (pACC) and their corresponding total forms were determined by Western blot. β-tubulin (β-Tub) serves as a loading control.
